# Theoretical Guidelines for Electrochemical C–F Bond Cleavage in Perfluorobutanoic Acid Using Transition Metal Catalysts

**DOI:** 10.1021/acsomega.6c00218

**Published:** 2026-03-05

**Authors:** Chi Ho Lee, Jay Liu, Joseph Sang-Il Kwon

**Affiliations:** † Artie McFerrin Department of Chemical Engineering, 14736 Texas A&M University, College Station, Texas 77845, United States; ‡ 34998 Pukyong National University, 45, Yongso-ro, Nam-Gu, Busan 48513, Republic of Korea; § William G. Lowrie Department of Chemical and Biomolecular Engineering, 2647 The Ohio State University, 151 W. Woodruff Avenue, Columbus, Ohio 43210, United States

## Abstract

Per- and polyfluoroalkyl substances (PFAS) are persistent pollutants with highly stable carbon–fluorine bonds, which makes catalytic degradation difficult. Among various catalytic strategies, electrochemical reduction has emerged as a practical alternative because it promotes C–F cleavage and H/F exchange. Transition metals (TMs) are particularly attractive for this process, since their conductivity and d-orbitals facilitate electron transfer into the C–F bond. Yet many theoretical studies overlook essential electrochemical factors; these include the hydrogen evolution reaction, surface oxidation, fluorine poisoning, and physisorption exclusion, and neglecting them limits realistic assessment of TM catalysts for PFAS degradation. Consequently, no theoretical framework exists to systematically screen catalysts under such rigorous constraints. To bridge this gap, we developed a theoretical screening protocol and applied it to 27 TMs, evaluating 81 surface facets. Specifically, this approach evaluates the viability of each metal surface for the electrochemical C–F cleavage in perfluorobutanoic acid (PFBA), chosen as a representative short-chain PFAS. Our screening incorporates all four essential electrochemical criteria, enabling the identification of 14 promising TM surface facets that satisfy the demanding requirements for PFBA degradation. An interesting trend emerging from this screening is that the difficulty of C–F cleavage is closely tied to how effectively electron density accumulates on the reacting carbon site. As defluorination progresses, later cleavage steps show sharply reduced charge transfer and correspondingly higher reaction free energies. This relationship suggests that a simple electronic descriptor can anticipate when C–F cleavage becomes energetically demanding.

## Graphical abstract

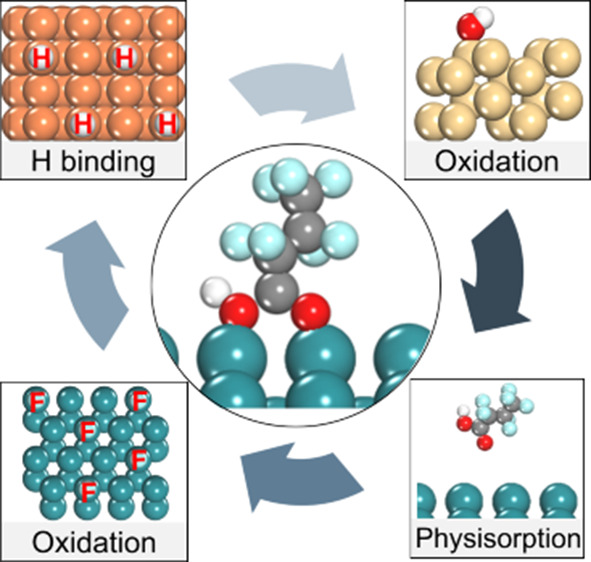

## 1 Introduction

Per- and polyfluoroalkyl substances (PFAS) comprise a large group of synthetic chemicals extensively used in industrial processes and consumer products such as firefighting foams, nonstick coatings, textiles, and food packaging due to their unique chemical stability and resistance to degradation.^1,2^ However, this stability leads to significant environmental persistence, resulting in widespread contamination in water bodies, soil, wildlife, and even humans.^3,4^ Recognizing their potential health hazards, including cancer, developmental effects, and immune dysfunction, global and national regulatory bodies have enacted stringent controls. For instance, the U.S. Environmental Protection Agency (EPA) recently proposed maximum contaminant levels as low as 4 ppt for specific PFAS such as PFOA and PFOS in drinking water.^5^ The chemical resistance of PFAS originates from the exceptional strength of their carbon–fluorine (C–F) bonds, which possess bond dissociation energies around 115–130 kcal/mol, substantially higher than typical C–H or C–C bonds.^6,7^ Because of this bond strength, many approaches have been explored to degrade PFAS. These include high-temperature incineration, advanced oxidation, reductive treatments, and various thermos-, photo-, sono- and electrochemical methods.^8–12^

Among these strategies, electrochemical approaches have received growing attention because they can be implemented in simple reactor setups, operate at relatively low cost, and can be powered by renewable electricity.^13–16^ In particular, electrochemical reduction has gained attention as a practical alternative that promotes C–F bond cleavage and hydrogen/fluorine (H/F) exchange reactions, thereby forming less-fluorinated products. Mechanistically, electrochemical reduction can proceed through two representative pathways. In the direct pathway, PFAS first adsorbs on the electrode surface and then undergoes dissociative electron transfer.^13^ This step can efficiently break the C–F bond, but doing so still requires sufficient energy input, accessible electrode potentials, and suitable catalyst materials. In contrast, the indirect pathway couples electron injection into the C–F bond with proton transfer from interfacial water or hydronium, so that H/F exchange yields a C–H product while F^–^ is released.^13^ Despite these differences, both pathways share two requirements: rapid electron transfer to the adsorbed PFAS and an electrode surface that couples electronically to the C–F bond. Among the major electrode materials, transition metal (TM) electrodes are a practical choice because they satisfy these two needs.^16^ First, metallic TMs offer high electrical conductivity, which enables fast electron transfer from the electrode to the adsorbed PFAS. Second, their tunable d-states can couple with PFAS molecular orbitals to assist C–F cleavage, as suggested by theory^17–20^ and supported by recent cathodic experiments.^21–23^ This interaction focuses on the antibonding σ* orbital, which is empty and able to accept electrons. When PFAS adsorbs on TM surfaces, the d-orbitals of TM efficiently interact with this antibonding orbital, creating a pathway for electron transfer. Under an applied cathodic potential, electrons on TM surfaces are injected into the antibonding orbitals, and once electron filled antibonding states, this orbital can weaken the C–F bond. As a result, the reaction energy required for bond cleavage decreases, enabling stepwise defluorination.^24–28^ Altogether, TMs are effective for PFAS reduction under ambient conditions because their metallic property allows rapid electron transfer, and their orbital interaction with PFAS enables electrons to occupy the antibonding state that weakens the C–F bond.

While these advantages make TMs promising for cathodic PFAS reduction, they introduce a clear trade-off with side reactions such as water reduction and the hydrogen evolution reaction (HER), which consumes electrons and occupies surface sites that would otherwise support PFAS adsorption and defluorination. To see how this trade-off appears in practice, mechanistic studies on diverse TMs (such as Au, Pt, Rh, and Ni) reveal a distinct PFAS reduction process that involves a defined formal potential and a concerted two-electron step. These results clarify where the reduction sites lie relative to the HER region and highlight why separating these potential windows is essential for observing PFAS reduction clearly.^13,17,29,30^ When HER dominates the reductive process, most of the supplied electrons are diverted to hydrogen gas formation rather than to PFAS. As a result, PFAS adsorption is hindered and its reduction becomes difficult to detect. Further, increasing the electrolyte alkalinity raises the HER overpotential, which suppresses hydrogen formation and allows the voltammetric signal of PFAS reduction to become visible on TM electrodes. In line with this, reviews^13,17,29,30^ on electrochemical PFAS reduction emphasize that HER should be evaluated under the same conditions, since the balance between H_2_ evolution and hydrodefluorination depends on the applied potential and the interfacial environment.

Despite how central HER is in experiments, many theoretical studies still do not clearly treat it, leaving a gap between what is measured and what is modeled. In practice, many calculations assume a clean and static TM surface and then analyze PFAS adsorption, a single C–F bond cleavage step, and simple links to metal electronic states, without accounting for competing hydrogen adsorption. As a result, the theoretical model overlooks the experimental reality that HER diverts electrons and occupies the surface sites required for PFAS adsorption and subsequent C–F activation. Accordingly, our screening uses DFT-derived descriptors to quantify competition between HER and PFAS reduction by comparing the potential to form adsorbed hydrogen with the potential at which PFAS adsorbs, and its C–F bond is activated. Specifically, if hydrogen binding occurs at less negative potentials or is substantially stronger than PFAS adsorption, we investigate the material as HER-prone and exclude it from the shortlist, and this criterion would serve as a minimal safeguard that removes candidates where competition is severe, even before diverse electrochemical environments are considered.

With this competition-aware screen in place, we select PFBA as a short-chain representative PFAS molecule to verify the utilization of the electrochemical reductive process. Further, we systematically investigate the H/F exchange pathways across 27 TMs by evaluating 81 surface facets across face-centered-cubic (FCC), body-centered-cubic (BCC), and hexagonal-close-packed (HCP) phases. By shortlisting all candidates based on strict criteria for competitive HER as well as diverse electrochemical factors, we identify 14 promising TM-facet combinations capable of effectively cleaving C–F bonds of PFBA under practical electrochemical conditions, with Cr(100) emerging as the top performer in our assessment. Beyond a simple ranking, this work sets a reference framework that can guide both theory and experiment toward rational design of TM-based catalysts. With this framework, we seek to provide usable theoretical insight for catalyst selection and to accelerate the discovery and application of effective electrocatalysts for PFAS degradation.

## 2 Materials and Methods

All ab initio calculations were performed with the Vienna Ab initio Simulation (VASP 5.4.4).^31^ We considered the Perdew–Burke–Ernzerhof (PBE)^32^ exchange–correlation functional using the projector augmented wave (PAW) method^33^ with a generalized gradient approximation (GGA)^32^ to accurately describe chemisorption on the surface. The Monkhorst–Pack^34^ *k*-point grid was used, and maximum symmetry was applied to reduce the number of *k*-points in all calculations. A plane-wave cutoff energy of 500 eV was used. Lattice constants and internal atomic positions were optimized until the residual forces became less than 0.02 eV/Å. Spin polarization and dipole correction were also included to decouple the electrostatic interaction between periodically repeated surface systems. All slab models were built with at least four atomic layers to capture surface relaxation while preserving bulk-like character; the bottom half of each slab was held fixed during optimization, and the remaining layers were fully relaxed. The vacuum slab space of a unit cell in the *z*-direction was set to 20 Å to avoid interactions between layers. Moreover, *k*-points were sampled using a 10 × 10 × 1 Monkhorst–Pack mesh.^34^ Bader charge analysis was performed using grid-based charge density decomposition, as developed by Henkelman et al.^35^ Long range dispersion interactions between PFBA and the transition metal surfaces were included using the DFT-D3 method with Becke Johnson damping (IVDW = 12).^36,37^

## 3 Results and Discussion

### 3.1 Construction of TM Surface Set Using Four Electrochemical Screening Criteria

We first defined a consistent computational framework designed to compare TM surfaces under consistent electrochemical conditions. The goal was to create a reference data set broad enough to capture periodic trends while still being systematic for PFBA defluorination analysis. To this end, we assembled a surface set covering 27 TMs and 81 low-index facets (Figure 1). Here, TMs were categorized according to their most stable bulk phases, FCC (red), HCP (green), and BCC (blue), so that the influence of lattice type could be evaluated explicitly. The FCC group includes Ni, Cu, Rh, Pd, Ag, Ir, Pt, and Au; the HCP group includes Sc, Ti, Zr, Hf, Tc, Ru, Re, Os, Co, Zn, and Cd; and the BCC group includes Y, V, Cr, Fe, Nb, Mo, Ta, and W. For each lattice, we selected only nonduplicate surface facets: FCC(100), (110), (111); HCP(0001), (1010), (1011); BCC(100), (110), (111). This organization corresponds to the top panel of Figure 1 and defines the reference set within which PFBA adsorption and C–F bond dissociation are evaluated. To ensure structural accuracy while allowing surface relaxation, all surface structures were constructed with at least four atomic layers. The lower half of each surface was fixed to preserve bulk-like character, while the upper layers were fully relaxed to allow surface reconstruction. Representative side- and top-view geometries for each lattice family are shown in the lower panels of Figure 1, providing a clear view of the atomic arrangements in the selected facets. By enforcing a uniform surface setup across all cases, we minimized size-related artifacts and ensured that the comparisons reflect only the intrinsic effects of the metal identity and surface orientation. This consistency provides a reliable baseline for evaluating how PFBA molecules interact with different TM surfaces.

**Figure 1:**
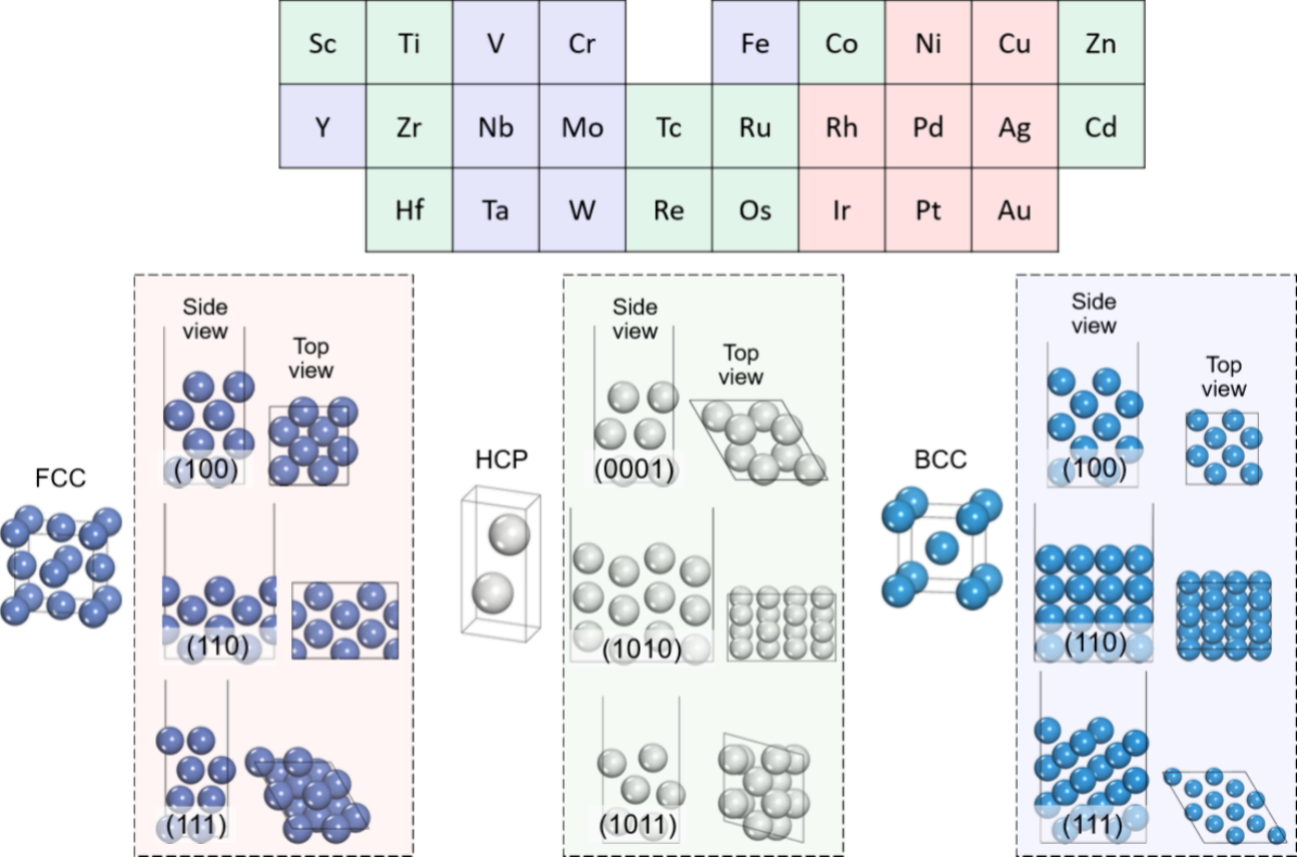
Surface library used in this work. Twenty-seven transition metals grouped by bulk lattice (FCC-red, HCP-green, and BCC-blue) combined with nonduplicate low-index facets yield 81 surface facets: FCC(100), FCC(110), FCC(111), HCP(0001), HCP(1010), HCP(1011), BCC(100), BCC(110), and BCC(111). Each surface has ≥4 layers with the bottom half fixed; side and top views illustrate representative geometries.

With this surface set in place, our first task is to evaluate whether PFBA can effectively adsorb on each TM surface under electrochemical conditions and proceed toward subsequent C–F bond cleavage. However, several competing processes can interfere either before PFBA binds or during its reaction, reducing the likelihood of successful defluorination. To account for these challenges, we introduce four exclusion criteria, grouped into two categories. The first category concerns processes that hinder PFBA adsorption: (a) hydrogen adsorption, which can occupy surface sites prior to PFBA arrival, (b) surface oxidation, where oxygenated species reduce adsorption availability, and (c) physisorption of PFBA, which indicates nonbonding physical interaction with the surface. The second category includes processes that interfere after adsorption, particularly during C–F bond cleavage: (d) fluorine poisoning, where released F atoms remain bound and deactivate nearby sites. If any one of these four criteria is more favorable than PFBA binding or reaction, the surface is removed from further consideration. Each is described in detail below.

#### 3.1.1 Category 1: Hindrance to PFBA Adsorption

a. Hydrogen adsorption: On TM surfaces, hydrogen binding from aqueous electrolytes can occur readily, even at electrode potentials much less than those required for PFBA binding and reduction. This means that long before PFBA can adsorb and begin C–F bond cleavage, the surface may already be covered by hydrogen atoms generated through water reduction. These adsorbed hydrogen atoms interfere with PFBA binding and reduction in two important ways. First, they occupy active sites that would otherwise bind PFBA, physically blocking the molecule from anchoring to the TM surfaces. Second, they can consume electrons that are needed for the reductive process of PFBA already attached to the surface. Consistent with this view, recent electrochemical experiments have already identified hydrogen evolution as a key competing pathway that must be carefully managed in PFAS reduction systems.^13,17,29,30^ Because of this, we exclude any TM surface where hydrogen binds more strongly than PFBA.
b. Surface oxidation: In addition to hydrogen binding, another factor that can interfere with PFBA binding is the accumulation of oxygenated species on the TM surface. While the overall process is conducted under cathodic conditions, TM surfaces can still interact with oxygen-containing species present in the aqueous electrolyte. Even at moderately negative potentials, adsorbates such as atomic oxygen (O*), hydroxyl groups (OH*), and water molecules (H_2_O*) can bind to the TM surface. This does not necessarily mean full surface oxidation is taking place, but partial coverage by these species can reduce the number of active sites available for PFBA binding. Moreover, under more negative potentials, surface reconstruction or persistent site blocking may occur due to strong interaction with oxygenated species (O*, OH*, and H_2_O*). Because of this, we exclude any TM surface where oxygenated species bind more than PFBA.
c. Physisorption of PFBA: In some cases, PFBA may adsorb on a TM surface only through weak van der Waals forces without forming significant chemical bonds. When this occurs, the electronic coupling between the surface and PFBA is too weak to enable efficient electron transfer. As a result, electrons cannot populate the antibonding orbitals of the C–F bond, and reductive cleavage becomes unlikely. Such physisorption can result not only from poor electronic interaction between the surface and PFBA, but also from steric hindrance, where the shape or orientation of the molecule prevents it from approaching the surface closely. Therefore, surfaces that allow only weak physisorption are excluded from further consideration, as they are unlikely to support effective C–F bond cleavage.

#### 3.1.2 Category 2: Interference with C–F Bond Cleavage

d. Fluorine poisoning: Once PFBA is adsorbed onto the TM surface and undergoes initial C–F bond cleavage, a fluorine atom may detach and remain on the surface. This adsorbed fluorine can block nearby active sites, preventing further approach and reaction of the PFBA molecule. More critically, these fluorine atoms can draw electron density away from the already adsorbed PFBA, limiting the availability of electrons needed for the next C–F cleavage. As a result, fluorine accumulation on the TM surface not only reduces the number of available reaction sites but also directly impairs the electron-driven defluorination process. To avoid such interference, we exclude TM surfaces predicted to retain fluorine strongly after the first cleavage.

Using the metrics in Tables 1 and S1, we applied the four criteria shown in Figure 2 and progressively narrowed the initial set of 81 metal-facet pairs. Although the four criteria are described sequentially for clarity, all metal-facet combinations were evaluated against each criterion independently. Any surface that failed even one criterion was removed from consideration. First, the criterion of competitive hydrogen binding (HER) removes Cr(110), Fe(110), Co(1010), Co(1011), Re(0001), Re(1010), Re(1011), Os(0001), and Os(1010). Second, the criterion of surface oxidation excludes V(110), Ni(100), Ni(110), Ni(111), Ru(0001), Ru(1010), Ru­(1011), Ir(100), Ir(110), and Ir(111), because O*, OH*, and H_2_O* binding becomes thermodynamically favorable at potentials lower than those required for PFBA adsorption, leading to site blocking and surface reconstruction. Third, the criterion of fluorine poisoning eliminates Y(0001), Y(1010), Y(1011), Zr(0001), Zr(1010), Zr(1011), Nb(100), Nb(110), Nb(111), Mo(100), Mo(111), Tc(0001), Tc(1010), Tc(1011), Hf(0001), Hf(1010), Hf(1011), Ta(100), Ta(110), Ta(111), W(100), W(111), and Os(1011), since strongly bound F* released after C–F cleavage persistently deactivate the TM surfaces. Finally, the criterion of weak physisorption removes Sc(1010); Cu(100) and Cu(111); Zn(0001), Zn(1010), Zn(1011); Rh(100), Rh(110), Rh(111); Pd(100), Pd(110), Pd(111); Ag(100), Ag(110), Ag(111); Cd(0001), Cd(1010), Cd(1011); Pt(100), Pt(111); and Au(100), Au(110), Au(111), since insufficient binding prevents efficient electron transfer and makes reductive C–F cleavage improbable. This parallel application of the four criteria ensures that only robust candidates, meaning surfaces that satisfy all constraints simultaneously, advance to the next stage. As a result of this comprehensive screening, 14 metal-facet combinations remain that satisfy all four criteria and thus represent plausible candidates for supporting potentially favorable PFBA adsorption and C–F bond cleavage under aqueous electrochemical conditions. These include Sc(0001), Sc(1011); Ti(0001), Ti(1010), Ti(1011); V(100), V(111); Cr(100), Cr(111); Fe(100), Fe(111); Co(0001); Mo(110); and W(110). Specifically, Figure 3 summarizes the PFBA adsorption free energies for these candidates. Most surfaces bind PFBA exothermically, suggesting that the molecule can stably adsorb and remain on the surface as a thermodynamically favorable state. However, adsorption is only the initial requirement for effective defluorination, and it does not guarantee that PFBA can proceed to bond cleavage efficiently.

**Figure 2:**
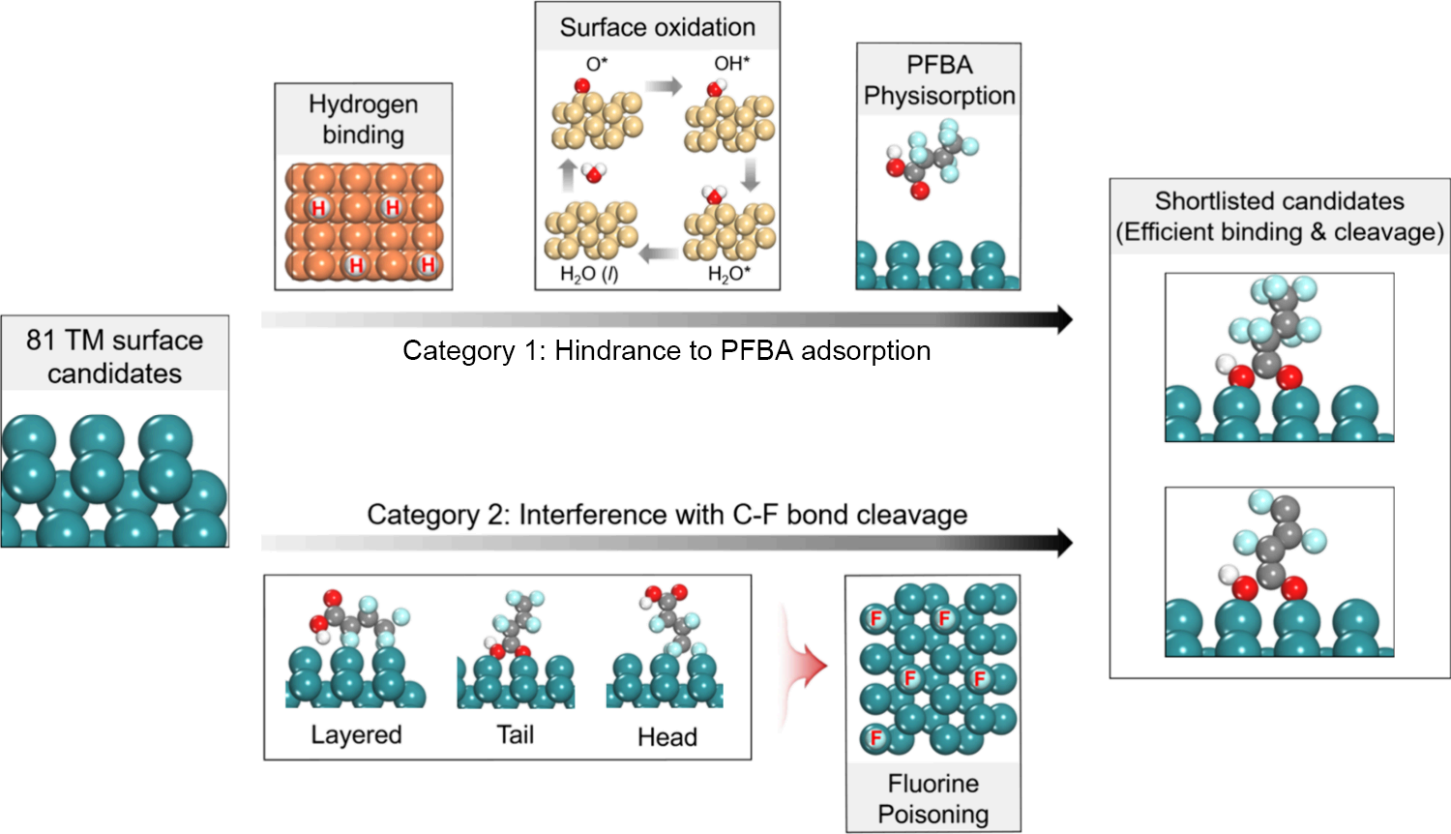
Four exclusion criteria, grouped into two categories to shortlist 81 TM surfaces. HER competition: strong H binding blocks PFBA adsorption. Surface oxidation: O, OH*, and H_2_O* formation deactivates sites. Physisorption of PFBA: surfaces on which PFBA binds only weakly are removed prior to C–F scission analysis. Fluorine poisoning: adsorption configurations that promote F* accumulations are excluded.

**Table 1:** Adsorption Free Energy of PFBA (Δ*G*_PFBA_) and Screening Outcome for All 81 Transition Metal Surface Facets Considered in This Work^a^

4 period	Δ*G*_PFBA_	screening outcome
Sc(0001)	–1.98	robust candidate
Sc(1010)	–4.57	excluded: physisorption of PFBA
Sc(1011)	–1.23	robust candidate
Ti(0001)	–0.88	robust candidate
Ti(1010)	–1.79	robust candidate
Ti(1011)	–0.88	robust candidate
V(100)	–0.57	robust candidate
V(110)	–1.60	excluded: surface oxidation
V(111)	–1.03	robust candidate
Cr(100)	–0.64	robust candidate
Cr(110)	–0.25	excluded: hydrogen adsorption
Cr(111)	0.11	robust candidate
Fe(100)	–3.34	robust candidate
Fe(110)	0.35	excluded: hydrogen adsorption
Fe(111)	–1.34	robust candidate
Co(0001)	0.13	robust candidate
Co(1010)	0.16	excluded: hydrogen adsorption
Co(1011)	0.27	excluded: hydrogen adsorption
Ni(100)	0.29	excluded: hydrogen adsorption and surface oxidation
Ni(110)	0.16	excluded: surface oxidation
Ni(111)	0.43	excluded: hydrogen adsorption and surface oxidation
Cu(100)	0.47	excluded: surface oxidation and physisorption of PFBA
Cu(110)	0.43	excluded: hydrogen adsorption and surface oxidation
Cu(111)	0.57	excluded: hydrogen adsorption, surface oxidation, and physisorption of PFBA
Zn(0001)	–0.73	excluded: physisorption of PFBA
Zn(1010)	0.25	excluded: surface oxidation and physisorption of PFBA
Zn(1011)	–1.41	excluded: surface oxidation and physisorption of PFBA

5 period	Δ*G*_PFBA_	screening outcome
Y(0001)	–0.29	excluded: hydrogen adsorption and fluorine poisoning
Y(1010)	0.93	excluded: hydrogen adsorption and fluorine poisoning
Y(1011)	0.92	excluded: hydrogen adsorption and fluorine poisoning
Zr(0001)	–0.29	excluded: hydrogen adsorption and fluorine poisoning
Zr(1010)	0.52	excluded: hydrogen adsorption and fluorine poisoning
Zr(1011)	–1.50	excluded: fluorine poisoning
Nb(100)	0.70	excluded: hydrogen adsorption and fluorine poisoning
Nb(110)	0.66	excluded: hydrogen adsorption and fluorine poisoning
Nb(111)	0.71	excluded: fluorine poisoning
Mo(100)	0.59	excluded: fluorine poisoning
Mo(110)	–0.15	robust candidate
Mo(111)	0.92	excluded: fluorine poisoning
Tc(0001)	0.41	excluded: surface oxidation and fluorine poisoning
Tc(1010)	–0.33	excluded: hydrogen adsorption and fluorine poisoning
Tc(1011)	0.80	excluded: fluorine poisoning
Ru(0001)	–1.14	excluded: physisorption of PFBA
Ru(1010)	0.19	excluded: hydrogen adsorption and surface oxidation
Ru(1011)	0.27	excluded: hydrogen adsorption and surface oxidation
Rh(100)	0.28	excluded: surface oxidation and physisorption of PFBA
Rh(110)	0.13	excluded: hydrogen adsorption and surface oxidation
Rh(111)	0.38	excluded: surface oxidation and physisorption of PFBA
Pd(100)	0.40	excluded: surface oxidation and physisorption of PFBA
Pd(110)	0.32	excluded: hydrogen adsorption and surface oxidation
Pd(111)	0.45	excluded: surface oxidation and physisorption of PFBA
Ag(100)	0.58	excluded: surface oxidation and physisorption of PFBA
Ag(110)	0.56	excluded: hydrogen adsorption, surface oxidation, and physisorption of PFBA
Ag(111)	0.68	excluded: surface oxidation and physisorption of PFBA
Cd(0001)	0.82	excluded: surface oxidation and physisorption of PFBA
Cd(1010)	1.03	excluded: surface oxidation and physisorption of PFBA
Cd(1011)	0.89	excluded: surface oxidation and physisorption of PFBA

6 period	Δ*G*_PFBA_	screening outcome
Hf(0001)	–0.76	excluded: fluorine poisoning
Hf(1010)	–5.06	excluded: fluorine poisoning
Hf(1011)	–2.47	excluded: fluorine poisoning
Ta(100)	–0.81	excluded: hydrogen adsorption and fluorine poisoning
Ta(110)	–4.19	excluded: hydrogen adsorption and fluorine poisoning
Ta(111)	–1.69	excluded: fluorine poisoning
W(100)	–3.63	excluded: hydrogen adsorption and fluorine poisoning
W(110)	–0.31	robust candidate
W(111)	–2.57	excluded: fluorine poisoning
Re(0001)	–0.55	excluded: hydrogen adsorption
Re(1010)	0.03	excluded: hydrogen adsorption and surface oxidation
Re(1011)	0.14	excluded: surface oxidation
Os(0001)	–0.08	excluded: hydrogen adsorption and surface oxidation
Os(1010)	–0.02	excluded: hydrogen adsorption and surface oxidation
Os(1011)	–1.94	excluded: fluorine poisoning
Ir(100)	0.24	excluded: hydrogen adsorption and surface oxidation
Ir(110)	0.28	excluded: hydrogen adsorption and surface oxidation
Ir(111)	0.55	excluded: surface oxidation and physisorption of PFBA
Pt(100)	0.45	excluded: surface oxidation and physisorption of PFBA
Pt(110)	0.27	excluded: hydrogen adsorption and surface oxidation
Pt(111)	0.51	excluded: surface oxidation and physisorption of PFBA
Au(100)	0.63	excluded: surface oxidation and physisorption of PFBA
Au(110)	0.67	excluded: hydrogen adsorption, surface oxidation, and physisorption of PFBA
Au(111)	0.68	excluded: surface oxidation and physisorption of PFBA

a The screening outcome summarizes whether each surface satisfies the four electrochemical criteria (hydrogen adsorption, surface oxidation, physisorption of PFBA, and fluorine poisoning). Facets that satisfy all four criteria are labeled “robust candidate”, whereas facets that fail one or more criteria are labeled “excluded” followed by the relevant criteria. Detailed reaction free energies for hydrogen adsorption and surface oxidation steps are provided in the Table S1.

**Figure 3:**
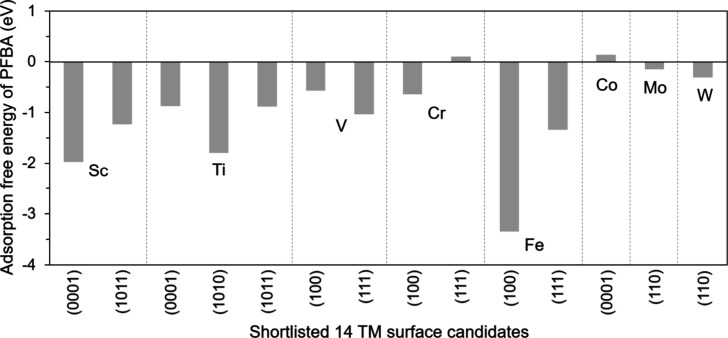
PFBA adsorption free energies for the 14 shortlisted transition-metal facets.

To assess this critical next step, we evaluate whether the adsorbed PFBA can undergo C–F bond cleavage through the H/F exchange mechanism on each of the remaining surfaces. The thermodynamic feasibility of this first cleavage event is analyzed in the next section.

### 3.2 Single C–F Bond Cleavage through H/F Exchange Mechanism

Figure 4a focuses on the proton driven H/F exchange mechanism. After PFBA adsorbs onto the TM surface, the first C–F bond cleaves, exposing a reactive carbon site. Because the C–F cleavage sends the electron pair with fluoride, the surface carbon is short by one electron. A proton then adds to this carbon, establishing the C–H bond framework while leaving the carbon electron-poor. Under cathodic bias, the electrode supplies the missing electron to complete the reduction, yielding a neutral and stable C–H bond. Based on this pathway, we evaluate the thermodynamics of the proton driven H/F exchange across the shortlisted TM surfaces to assess whether the first bond breaking step is accessible at experimentally relevant potentials.

**Figure 4:**
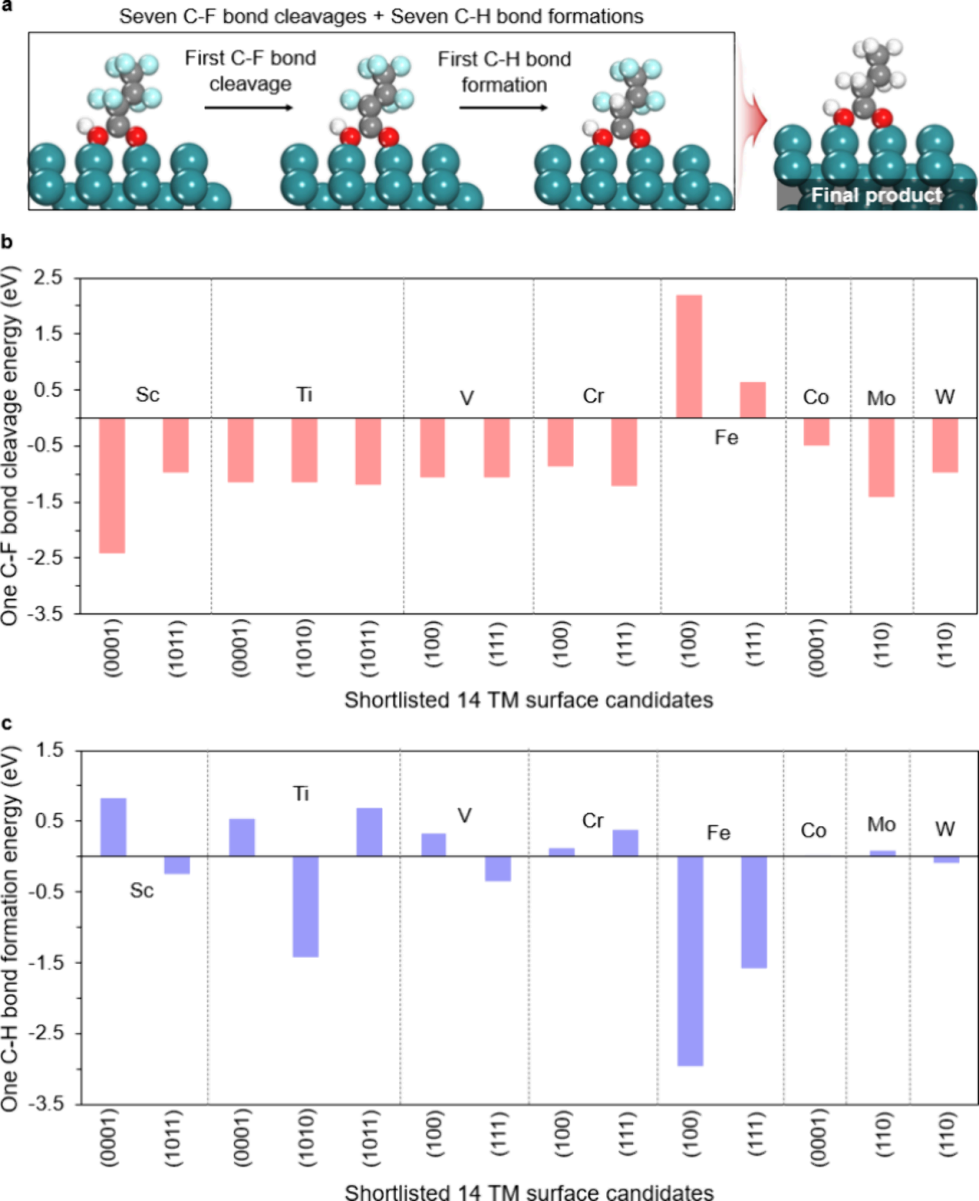
Single-step energetics after PFBA adsorption. (a) Scheme of initial C–F cleavage followed by C–H formation on a TM surface. (b) Free energy of the first C–F cleavage for the 14 shortlisted TM–facet pairs. (c) Free energy of C–H formation at the cleaved carbon for the same set. Energies referenced to the adsorbed state; V(110) was excluded due to surface oxidation occurring during the optimization.

Figure 4b shows the reaction free energies associated with the initial C–F bond cleavage across 14 TM surfaces, and most candidates exhibit negative values, indicating that the first C–F cleavage step is thermodynamically favorable. The values follow this order: Sc(0001), −2.42 eV < Mo(110), −1.42 eV < Cr(111), −1.22 eV < Ti(1011), −1.19 eV < Ti(1010), −1.15 eV < Ti(0001), −1.14 eV < V(100), −1.06 eV < V(111), −1.05 eV < W(110), −0.97 eV < Sc(1011), −0.97 eV < Cr(100), −0.86 eV < Co(0001), −0.49 eV < Fe(111), 0.64 eV < Fe(100), 2.20 eV.

To understand whether the reaction can proceed beyond cleavage, Figure 4c evaluates the thermodynamics of the subsequent hydrogenation step, in which a hydrogen fills the cleaved carbon site. In most cases, this step is either thermoneutral or slightly exergonic, implying that only a modest cathodic potential would be required to stabilize the product. The corresponding free energies follow this order: Fe(100), −2.95 eV < Fe(111), −1.58 eV < Ti(1010), −1.43 eV < V(111), −0.35 eV < Sc(1011), −0.25 eV < W(110), −0.09 eV < Co(0001), 0.02 eV < Mo(110), 0.09 eV < Cr(100), 0.13 eV < V­(100), 0.33 eV < Cr(111), 0.37 eV < Ti(0001), 0.54 eV < Ti(1011), 0.69 eV < Sc(0001), 0.83 eV. Taken together, these results reinforce the view that while PFBA adsorption provides a necessary starting point, the feasibility of C–F bond cleavage and the favorability of subsequent hydrogenation ultimately dictate whether defluorination can proceed efficiently. Having identified the fundamental understanding of the first cleavage step in initiating PFBA degradation, we now extend the analysis to multistep defluorination. In the next section, we evaluate how the energetics evolve with successive C–F bond cleavages and identify the potential thresholds associated with deeper reduction along the full reaction pathway.

### 3.3 Stepwise Defluorination: Potentials for Partial and Complete C–F Bond Cleavage

Building on the single-step results in Section 3.2, we now expand the analysis to a full defluorination sequence involving multiple C–F bond cleavages. For each intermediate along the pathway, all remaining fluorinated carbon sites are evaluated, and the most favorable position for the next cleavage is selected based on its reaction free energy. After each C–F bond is broken, we assess the thermodynamics of hydrogenation at the resulting carbon site, reflecting the H/F exchange mechanism under aqueous electrochemical conditions.

This iterative process continues until all seven fluorine atoms are removed. Specifically, we computed the full defluorination sequence set and assembled the results as complete free energy diagrams (FEDs) and potential determining steps (PDSs) for every surface in Figure S1, with the reaction free energies for each step and the hydrogenation data summarized in Tables S2 and S3. From this data set, the surface with the best performance is Cr(100), which gives the lowest overall limiting potential among the 14 candidates; its pathway is shown in Figure 5a. In this case, the PDS is the fifth C–F cleavage, which requires 1.35 V. Taken together, these comparisons show that the potential for complete defluorination of one PFBA molecule ranges from 1.35 to 3.04 V across the surfaces. They also show a consistent pattern: the highest barriers arise at C–F cleavages, whereas hydrogenation steps are comparatively mild.

**Figure 5:**
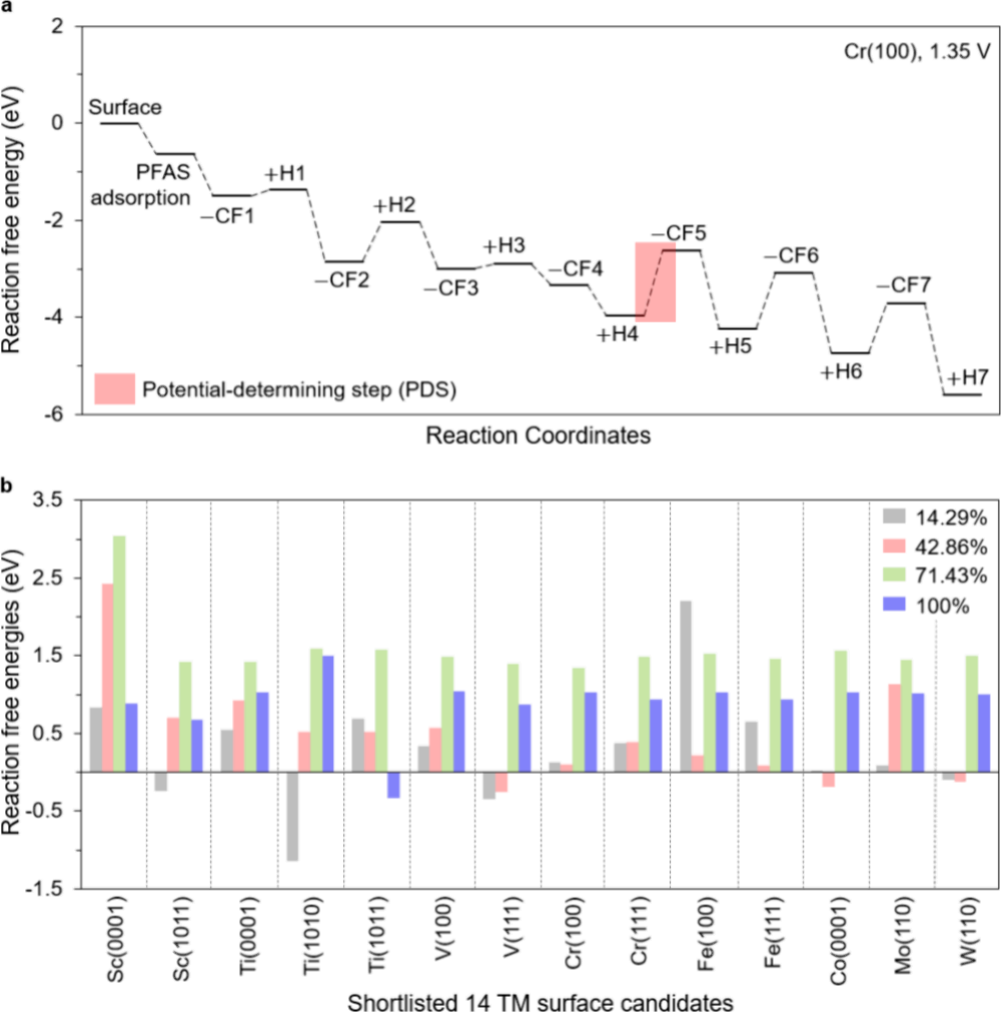
Sequential defluorination energetics and required potentials. (a) Free energy diagram (FED) for Cr(100) along the most favorable pathway: seven C–F cleavages (CF1–CF7) alternate with seven hydrogenations (H1–H7). The potential-determining step occurs at CF5, giving a required potential of 1.35 V. (b) Reaction free energies (eV) needed to reach four defluorination extents corresponding to 1, 3, 5, and 7 C–F cleavages (14.29, 42.86, 71.43, and 100%), evaluated for the 14 shortlisted TM–facet pairs.

Given the wide potential range for full defluorination, it is useful to ask how much conversion can be achieved at lower cathodic potential. This question is practically relevant because PFAS treatment is often implemented as a multi-stage process that combines cathodic reduction with complementary oxidation steps.^38–42^ In such systems, when some C–F bonds are converted to C–H, the chain becomes less electron poor because fluorine no longer pulls as strongly on electron density at those positions. In that relieved state, removing electrons and forming radicals would be easier for the oxidation process. This increase in electronic flexibility raises the polarizability of the carbon backbone and restores electron density to adjacent carbons and reduces the over polarization of nearby C–F, making those C–F bonds easier to cleave in water. Furthermore, the presence of C–H opens a new entry point for oxidants in water: hydroxyl radicals and anodic oxygen species can abstract H from C–H to generate a reactive carbon center on the chain. That center then reacts rapidly with oxygen species or generates H–F, which further weakens adjacent C–F and propagates additional bond breaking. In short, introducing C–H both softens fluorine driven electron withdrawal that stabilizes the perfluorinated chain and creates accessible H abstraction sites, so partially defluorinated intermediates proceed through oxidation much more readily than fully fluorinated ones. These practical considerations motivate reporting potentials for partial defluorination in addition to full conversion. Therefore, Figure 5b summarizes the potentials required to reach four levels of conversion corresponding to cleavage of one, three, five, and seven C–F bonds (14.29, 42.86, 71.43, and 100%). Specifically, two trends emerge. First, with the exception of Fe(100) and Sc(0001), most surfaces achieve the first cleavage near 0.5 V, and 12 candidates reach three cleavages below 1.0 V, with Cr(100) standing out at only 0.13 V for this stage. Second, the required potential increases sharply once five C–F bonds are cleaved (71.43% conversion). Overall, Figure 5 highlights three points: adsorption provides a stable starting state, the first few C–F cleavages are accessible at modest potentials, and the midsequence step, especially the fifth cleavage, sets the potential required for deep defluorination. Taken together, these trends indicate whether to aim for full cathodic conversion or to integrate partial defluorination into a multi-stage treatment that couples cathodic reduction with anodic oxidation. Full degradation remains the goal and is preferred if the cathode can easily overcome the fifth C–F cleavage barrier. When that is not feasible, it would be useful to set partial defluorination targets at the reported cathodic potentials for one, three, and five C–F cleavages and then design the anodic process around the C–H containing intermediates produced at the cathode.

### 3.4 Correlation between Reaction Free Energy and Charge at Reacting Carbon Sites

Because Cr(100) already showed the highest activity among the 14 TM surfaces, we now use this surface to understand why the fifth C–F cleavage becomes the PDS. For this purpose, we examined how much electron charge accumulates on the reacting carbon atom at each cleavage step using Bader charge analysis (Figure 6). For four representative C–F bond cleavage ratios (14.29, 42.86, 71.43, and 100%), it reports the reaction free energy required for the next C–F bond cleavage, as well as the Bader charge on the carbon atom where the cleavage occurs. The Bader charge serves as an indicator of how much electron density is concentrated at that site. The inset figures illustrate PFBA intermediates at each C–F bond cleavage ratio, showing the local environments of the reacting carbon atoms as C–F bonds are sequentially removed and C–H bonds are formed. Up to 42.86% C–F bond cleavage, the reaction free energies remain relatively low, which indicates that the first several C–F bonds can be cleaved with a relatively small free energy change and that electrons are transferred efficiently from the Cr(100) surface to the reacting carbon atoms. However, once the cleavage ratio reaches 71.43% (fifth C–F bond cleavage), the reaction free energy rises sharply and stays high at 100%. At these later steps, the reacting carbon atoms carry much less negative charge and are located farther from the surface, indicating poor electron supply and a weak reductive process. Based on this, we confirm a clear correlation between reaction free energy and the quantity of electron charge, and this correlation identifies the fifth C–F bond cleavage as the PDS. More broadly, this result suggests that Bader charge can be used as a simple descriptor for C–F bond cleavage. By first calculating the Bader charges of carbon atoms in a series of partially defluorinated structures, one can anticipate which cleavage steps will require large reaction free energies, and which step is likely to become the PDS. In future studies, this type of charge-based descriptor could be combined with reaction kinetic models to connect local electronic structure to observable reaction rates.^43–45^ Such an extension would help evaluate how PFAS defluorination proceeds under operating electrochemical conditions and how it competes with other interfacial reactions.

**Figure 6:**
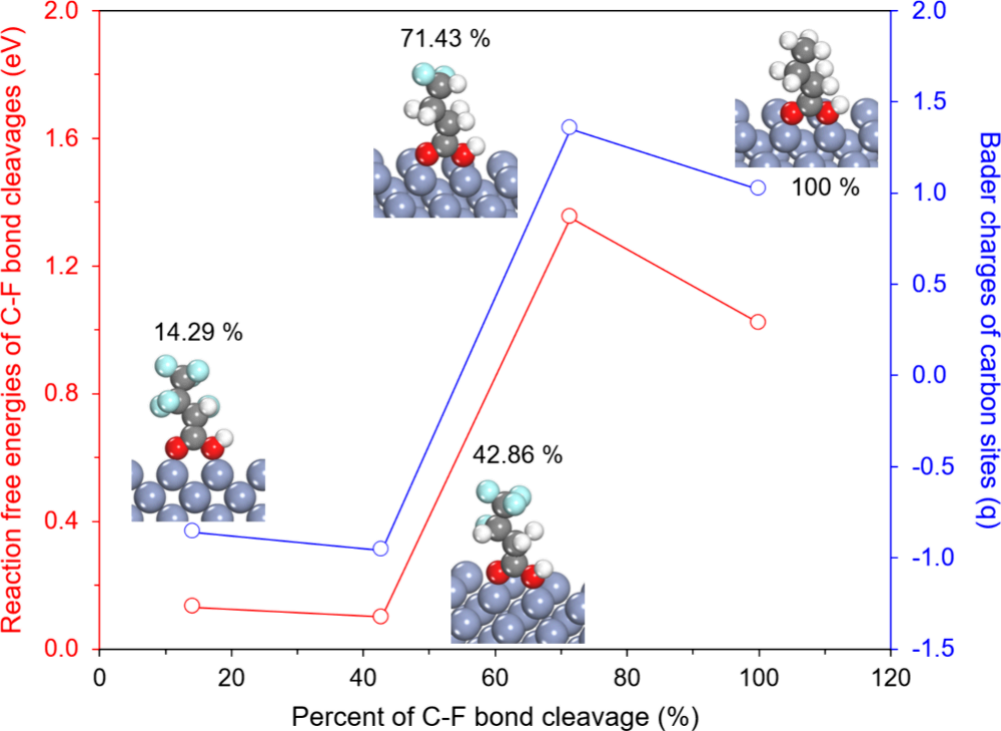
For PFBA adsorbed on the Cr(100) surface, reaction free energies for each C–F bond cleavage step (red, left axis) and the corresponding Bader charges on the carbon atoms (blue, right axis) as a function of the percentage of C–F bonds cleaved in PFBA. As the C–F bond cleavage ratio increases, the reaction free energy and the charge on carbon sites at different positions change in a correlated way, showing a sharp increase in the free energy at high cleavage ratios and a gradual loss of accumulated negative charge on the carbon atoms. The insets illustrate representative intermediates at 14.29, 42.86, 71.43, and 100% C–F cleavage with C–H formation.

## 4 Conclusions

This work sets out to build a theory-based foundation for selecting transition metal surfaces that can support electrochemical PFBA defluorination under realistic aqueous conditions. We first constructed a consistent surface set of 27 metals and 81 facets, and then applied four electrochemical exclusion criteria that capture key challenges in practice, namely competition from hydrogen adsorption, partial surface oxidation, fluorine poisoning, and weak physisorption of PFBA. This parallel filtering step reduced the initial set to 14 metal facet combinations that can both bind PFBA favorably and avoid these competing processes. Based on 14 TM candidates, analysis of the first C–F bond cleavage and the subsequent hydrogenation step showed that many candidates can initiate defluorination at modest cathodic potentials, and that hydrogenation steps are generally mild compared to C–F bond breaking. Extending this view to the full sequence of seven cleavages revealed a consistent pattern across the candidates. Adsorption provides a stable starting state, and the early C–F cleavages proceed at relatively low potentials. In contrast, a middle step, represented by the fifth cleavage, requires the highest potential and therefore limits how far defluorination can proceed under cathodic conditions. Among the shortlisted candidates, Cr(100) shows the lowest limiting potential, so we examined this surface in detail and found a strong correlation between the reaction free energy of each cleavage step and the electron charge accumulated on the reacting carbon. This trend suggests that a simple charge-based descriptor can anticipate which C–F cleavages will require large free energy changes and are likely to become PDS steps.

## Supplementary Material



## Notes

Calculated reaction free energies for PFAS degradation; and free energy diagrams (FED) for shortlisted TM surfaces (PDF)
