# Supplementary material for: Theoretical Guidelines for Electrochemical C–F Bond Cleavage in Perfluorobutanoic Acid Using Transition Metal Catalysts
Source: ACS Omega. 2026 Mar 5;11(10):16844–53. doi: 10.1021/acsomega.6c00218 (PMC13000578; doi:10.1021/acsomega.6c00218)
Supplement: Supplementary file 1 [file ao6c00218_si_001.pdf]

Theoretical Guidelines for Electrochemical C-F Bond Cleavage in perfluorobutanoic acid Using Transition Metal Catalysts

Chi Ho Lee[a], Jay Liu[b*], and Joseph Sang-Il Kwon[c*]

[a]*Artie McFerrin Department of Chemical Engineering, Texas A&M University, College Station, TX 77845 USA*

[b]*Pukyong National University, 45, Yongso-ro, Nam-Gu, Busan, 48513, Republic of Korea*

[c]*William G. Lowrie Department of Chemical and Biomolecular Engineering, The Ohio State University, 151 W. Woodruff Avenue, Columbus, OH 43210, USA*

*Co-corresponding authors *jayliu@pknu.ac.kr (Jay Liu) and kwon.677@osu.edu (Joseph Sang-Il Kwon)*

Table S1. Reaction free energies for PFBA adsorption (ΔG_{PFBA}), hydrogen adsorption relevisto HER (ΔG_{HER}), and three surface-oxidation steps ($\Delta G_{(OH^*\text{-}O^*)}$, $\Delta G_{(H2O^*\text{-}OH^*)}$, and $\Delta G_{(H2O\text{-}H2O^*)}$), where OH*, O*, and H_2O* denote adsorbed species and H_2O (l) denotes liquid water. Further, *, **, and *** indicate TM surfaces with physisorption of PFBA, F poisoning, and OH dissociation from PFBA followed by surface occupation, respectively.

4 Period	ΔG_{PFBA}	ΔG_{HER}	$\Delta G_{(OH^*\text{-}O^*)}$	$\Delta G_{(H2O^*\text{-}OH^*)}$	$\Delta G_{(H2O\text{-}H2O^*)}$
Sc(0001)	-1.98	-0.82	2.01	1.11	-0.45
Sc(1010)	-4.57*	-1.37	-0.73	1.02	-0.08
Sc(1011)	-1.23	-0.88	1.25	1.76	-0.45
Ti(0001)	-0.88	-0.60	0.41	1.59	0.46
Ti(1010)	-1.79	-0.88	-0.48	0.64	-0.27
Ti(1011)	-0.88	0.06	1.74	0.84	-0.44
V(100)	-0.57	0.04	-0.13	0.76	0.20
V(110)	-1.60***	-1.15	0.46	1.74	0.16
V(111)	-1.03	0.21	-0.49	0.47	0.45
Cr(100)	-0.64	-0.16	-0.08	0.54	0.27
Cr(110)	-0.25	-0.54	1.87	-0.21	0.63
Cr(111)	0.11	0.40	-0.33	0.32	0.20
Fe(100)	-3.34	0.06	-0.67	0.25	-0.08
Fe(110)	0.35	-0.08	0.92	-0.05	-0.02
Fe(111)	-1.34	-0.25	-0.78	0.44	-0.35
Co(0001)	0.13	0.45	3.30	2.53	-0.20
Co(1010)	0.16	-1.35	-1.00	0.02	-0.51
Co(1011)	0.27	-0.33	0.12	0.01	0.09
Ni(100)	0.29	0.16	-1.26	-0.61	0.05
Ni(110)	0.16	0.20	-1.91	0.20	-0.38
Ni(111)	0.43	-0.34	0.34	-0.73	-0.05
Cu(100)	0.47*	0.50	0.30	-0.50	-0.16
Cu(110)	0.43	0.40	-1.85	-0.48	-0.01
Cu(111)	0.57*	-0.16	0.07	-0.77	-0.44
Zn(0001)	-0.73*	0.26	-0.35	-0.28	-0.04
Zn(1010)	0.25*	0.36	-2.75	0.87	-0.31
Zn(1011)	-1.41*	0.55	-0.47	-2.30	1.87
5 Period	ΔG_{PFBA}	ΔG_{HER}	$\Delta G_{(OH^*\text{-}O^*)}$	$\Delta G_{(H2O^*\text{-}OH^*)}$	$\Delta G_{(H2O\text{-}H2O^*)}$
Y(0001)	-0.29**	-0.62	1.98	1.19	-0.36
Y(1010)	0.93**	-1.27	-0.86	1.37	-0.38
Y(1011)	0.92**	-0.76	1.61	1.09	0.27

Zr(0001)	-0.29**	-0.48	1.31	0.78	-0.48
Zr(1010)	0.52**	-0.82	-0.59	0.32	-0.38
Zr(1011)	-1.50**	-0.02	-0.33	0.39	-0.40
Nb(100)	0.70**	0.13	0.00	0.99	0.14
Nb(110)	0.66**	-0.79	-0.36	0.53	0.26
Nb(111)	0.71**	0.28	-0.45	0.62	0.28
Mo(100)	0.59**	-0.73	3.59	0.33	0.42
Mo(110)	-0.15	0.17	-0.06	0.06	0.16
Mo(111)	0.92**	0.20	-0.24	0.63	-0.22
Tc(0001)	0.41**	-0.22	-0.31	0.49	-0.60
Tc(1010)	-0.33**	-1.69	0.47	-0.39	-0.37
Tc(1011)	0.80**	-0.25	-0.09	-0.03	0.36
Ru(0001)	-1.14*	-0.47	-0.22	0.01	-0.35
Ru(1010)	0.19	-0.20	-0.39	0.30	0.05
Ru(1011)	0.27	-0.29	-0.23	-0.29	0.35
Rh(100)	0.28*	-0.18	-0.96	-0.59	-0.42
Rh(110)	0.13	-0.08	-0.92	-0.46	-0.44
Rh(111)	0.38*	-0.41	-1.14	-0.54	-0.58
Pd(100)	0.40*	0.15	-6.47	-0.75	4.56
Pd(110)	0.32	-0.02	-1.57	-0.76	0.00
Pd(111)	0.45*	-0.46	-1.62	-0.99	-0.11
Ag(100)	0.58*	0.63	-1.06	0.00	-0.24
Ag(110)	0.56*	0.73	-2.26	-0.88	-0.2
Ag(111)	0.68*	0.25	-0.81	-1.09	-0.24
Cd(0001)	0.82*	0.57	-0.53	-0.51	-0.27
Cd(1010)	1.03*	0.60	-2.07	-0.43	-0.35
Cd(1011)	0.89*	0.71	-0.77	-0.63	-0.37
6 Period	ΔG_{PFBA}	ΔG_{HER}	$\Delta G_{(OH*-O*)}$	$\Delta G_{(H2O*-OH*)}$	$\Delta G_{(H2O-H2O*)}$
Hf(0001)	-0.76**	0.09	1.34	1.12	-0.28
Hf(1010)	-5.06**	0.24	-0.50	1.26	-0.45
Hf(1011)	-2.47**	-0.13	-0.51	1.00	-0.20
Ta(100)	-0.81**	-1.00	-0.25	1.04	-0.55
Ta(110)	-4.19**	-0.88	-0.48	0.56	-0.37
Ta(111)	-1.69**	0.17	-0.60	0.69	-0.48
W(100)	-3.63**	-1.27	0.27	0.62	-0.38
W(110)	-0.31	-0.06	0.06	0.35	0.15
W(111)	-2.57**	-0.02	-0.26	0.72	-0.29
Re(0001)	-0.55	-0.81	-0.26	0.68	-0.40
Re(1010)	0.03	-0.32	-0.26	0.45	-0.81
Re(1011)	0.14	-0.17	-0.05	0.11	0.43

Os(0001)	-0.08	-0.36	-0.05	0.32	-0.43
Os(1010)	-0.02	-0.55	-0.10	0.25	-0.51
Os(1011)	-1.94**	-0.40	-0.01	-0.03	-0.45
Ir(100)	0.24	-0.51	-0.79	-0.55	0.18
Ir(110)	0.28	-0.36	-0.68	-0.38	-0.44
Ir(111)	0.55*	-0.34	-1.08	-0.50	-0.14
Pt(100)	0.45*	-0.35	-1.41	-0.85	-0.01
Pt(110)	0.27	-0.46	-1.29	-0.72	0.14
Pt(111)	0.51*	-0.39	-1.49	-1.01	-0.09
Au(100)	0.63*	0.27	-2.05	-1.05	-0.22
Au(110)	0.67*	0.28	-2.09	-1.09	-0.20
Au(111)	0.68*	0.16	-2.03	-1.33	-0.28

Table S2. Reaction free energies (eV) for sequential C–F bond cleavages on the shortlisted TM–facet surfaces. Values are listed for the first through seventh cleavages (CF1–CF7) along the most favorable pathway. * denotes surface oxidation occurring during the optimization.

**4 Peiord**	**First C-F cleavage**	**Second C-F cleavage**	**Third C-F cleavage**	**Fourth C-F cleavage**	**Fifth C-F cleavage**	**Sixth C-F cleavage**	**Seventh C-F cleavage**
Sc(0001)	-2.42	-4.00	-3.34	-3.16	-3.35	0.93	0.88
Sc(1011)	-0.97	-2.55	0.70	0.15	1.42	-0.98	0.68
Ti(0001)	-1.14	-1.63	0.92	0.74	1.42	1.31	1.02
Ti(1010)	-1.15	0.79	-1.88	-1.41	1.59	1.30	1.50
Ti(1011)	-1.19	-2.25	-1.42	-0.93	1.57	-0.15	-0.34
V(100)	-1.06	-2.54	-1.48	0.84	1.49	1.28	1.04
V(111)	-1.05	-2.57	-0.50	0.72	1.40	1.13	0.87
Cr(100)	-0.86	-1.47	-0.96	-0.43	1.35	1.15	1.02
Cr(111)	-1.22	0.18	-1.42	0.66	1.48	1.23	0.94
Fe(100)	2.20	1.19	-1.18	0.88	1.53	1.25	1.02
Fe(111)	0.64	0.93	-1.04	1.98	1.45	1.23	0.94
Co(0001)	-0.49	-0.86	-1.08	-0.15	1.56	1.33	1.03
**5 Peiord**	**First C-F cleavage**	**Second C-F cleavage**	**Third C-F cleavage**	**Fourth C-F cleavage**	**Fifth C-F cleavage**	**Sixth C-F cleavage**	**Seventh C-F cleavage**
Mo(110)	-1.42	-1.44	1.13	0.82	1.45	1.26	1.01
**6 Peiord**	**First C-F cleavage**	**Second C-F cleavage**	**Third C-F cleavage**	**Fourth C-F cleavage**	**Fifth C-F cleavage**	**Sixth C-F cleavage**	**Seventh C-F cleavage**
W(110)	-0.97	-1.91	-0.96	1.00	1.50	1.31	1.00

Table S3. Reaction free energies (eV) for sequential C–H bond formations on the shortlisted TM–facet surfaces. Values are listed for the first through seventh formations (CH1–CH7) along the most favorable pathway. * denotes surface oxidation occurring during the optimization.

**4 Peiord**	**First C-F cleavage**	**Second C-F cleavage**	**Third C-F cleavage**	**Fourth C-F cleavage**	**Fifth C-F cleavage**	**Sixth C-F cleavage**	**Seventh C-F cleavage**
Sc(0001)	0.83	3.00	2.43	2.42	3.04	-1.41	-4.22
Sc(1011)	-0.25	1.71	0.12	-2.70	-0.16	0.19	-1.27
Ti(0001)	0.54	0.68	-1.71	-1.73	-1.74	-1.74	-1.91
Ti(1010)	-1.43	-0.81	0.51	0.44	-1.89	-1.75	-2.42
Ti(1011)	0.69	1.61	0.52	-0.14	-1.84	-0.32	-0.54
V(100)	0.33	1.80	0.57	-1.92	-1.75	-1.78	-1.87
V(111)	-0.35	1.71	-0.25	-1.69	-1.65	-1.60	-1.72
Cr(100)	0.13	0.81	0.10	-0.64	-1.61	-1.65	-1.88
Cr(111)	0.37	-0.91	0.39	-1.71	-1.81	-1.69	-1.82
Fe(100)	-2.95	-1.91	0.21	-2.00	-1.75	-1.76	-1.88
Fe(111)	-1.58	-1.54	0.09	-3.08	-1.77	-1.71	-1.82
Co(0001)	0.02	0.20	-0.19	-0.92	-1.90	-1.76	-1.89
**5 Peiord**	**First C-F cleavage**	**Second C-F cleavage**	**Third C-F cleavage**	**Fourth C-F cleavage**	**Fifth C-F cleavage**	**Sixth C-F cleavage**	**Seventh C-F cleavage**
Mo(110)	0.09	0.33	-1.79	-1.97	-1.82	-1.75	-1.94
**6 Peiord**	**First C-F cleavage**	**Second C-F cleavage**	**Third C-F cleavage**	**Fourth C-F cleavage**	**Fifth C-F cleavage**	**Sixth C-F cleavage**	**Seventh C-F cleavage**
W(110)	-0.09	1.28	-0.13	-2.11	-1.85	-1.77	-1.91

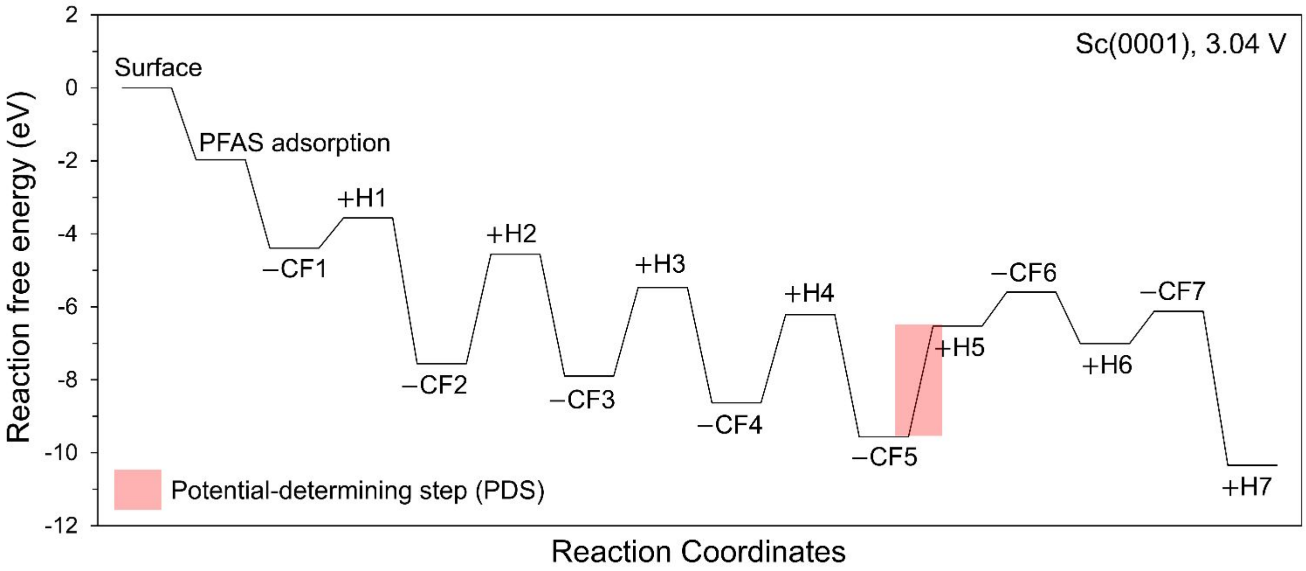
Sc(0001), 3.04 V
Reaction free energy (eV)
Reaction Coordinates
2
0
-2
-4
-6
-8
-10
-12
Surface
PFAS adsorption
−CF1
+H1
−CF2
+H2
−CF3
+H3
−CF4
+H4
−CF5
+H5
−CF6
+H6
−CF7
+H7
Potential-determining step (PDS)

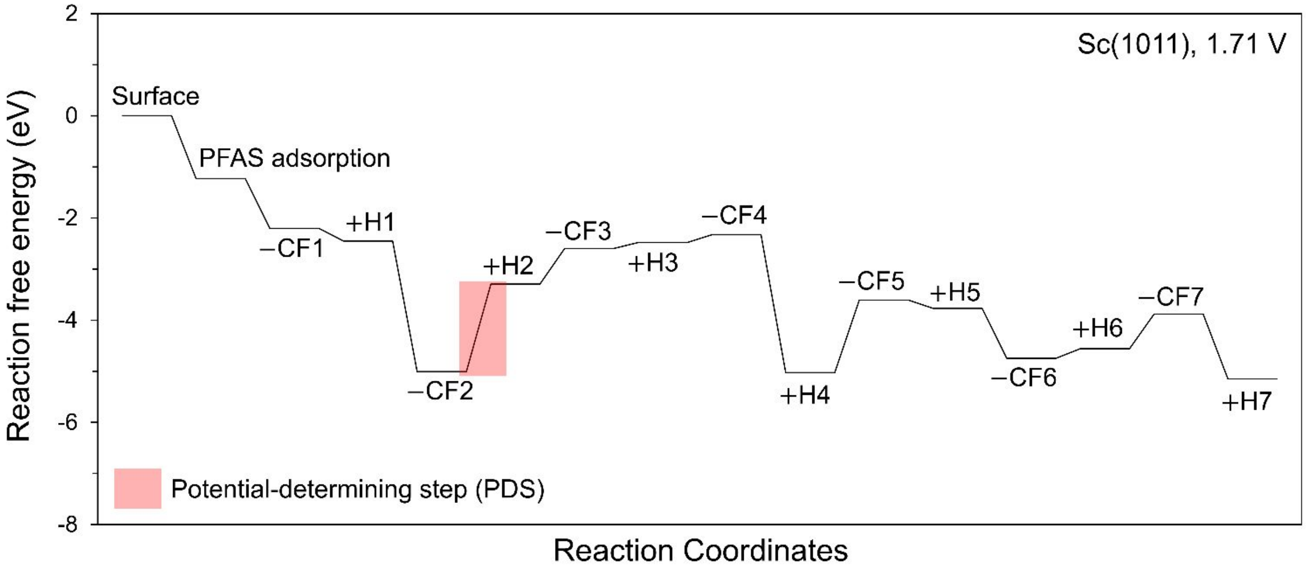
Sc(1011), 1.71 V
Reaction free energy (eV)
Reaction Coordinates
2
0
-2
-4
-6
-8
Surface
PFAS adsorption
−CF1
+H1
−CF2
+H2
−CF3
+H3
−CF4
+H4
−CF5
+H5
−CF6
+H6
−CF7
+H7
Potential-determining step (PDS)

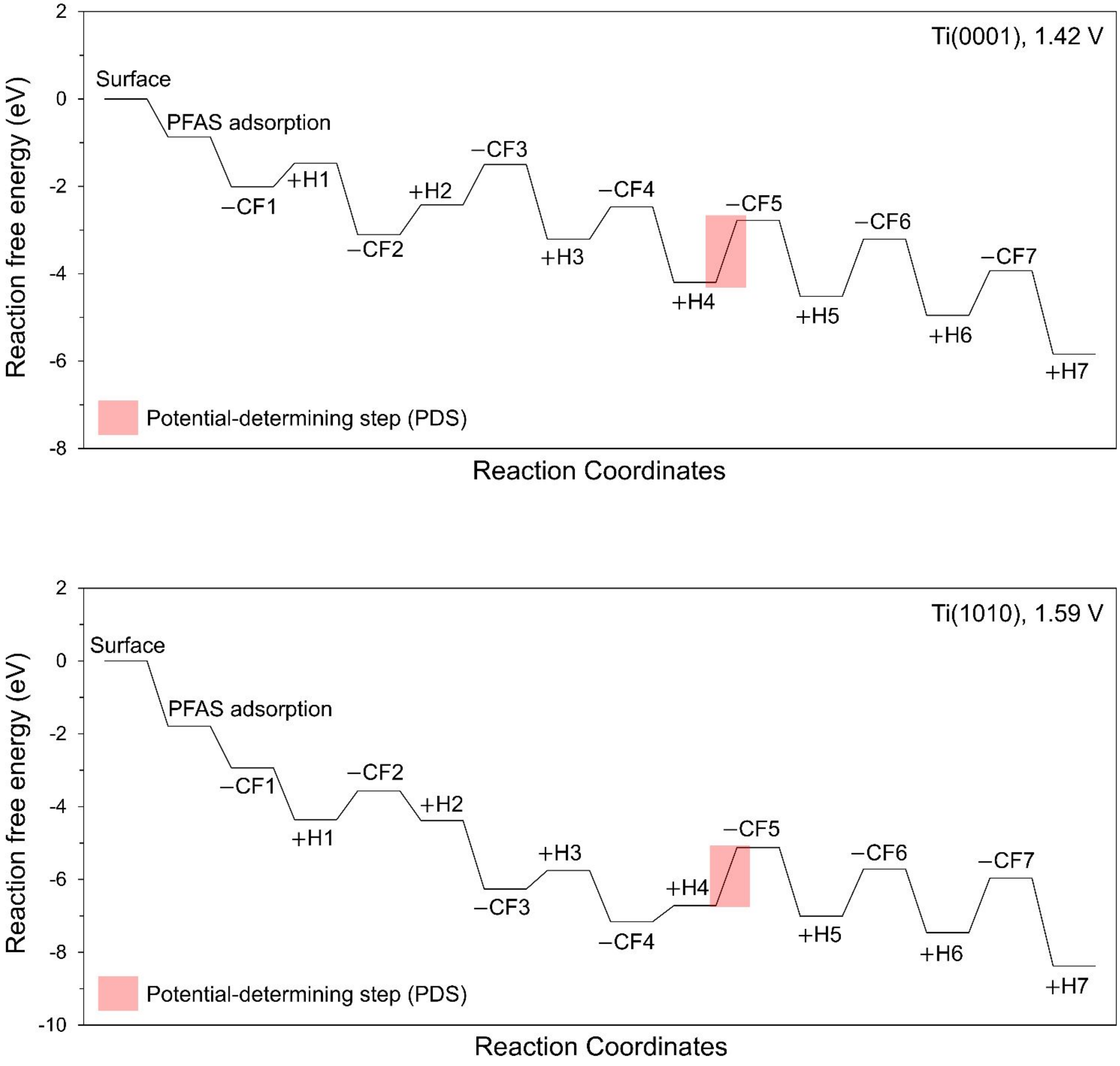
Ti(0001), 1.42 V
Reaction free energy (eV)
Reaction Coordinates
Surface
PFAS adsorption
−CF1
+H1
−CF2
+H2
−CF3
+H3
−CF4
+H4
−CF5
+H5
−CF6
+H6
−CF7
+H7
Potential-determining step (PDS)
2
0
-2
-4
-6
-8
Ti(1010), 1.59 V
Reaction free energy (eV)
Reaction Coordinates
Surface
PFAS adsorption
−CF1
+H1
−CF2
+H2
−CF3
+H3
−CF4
+H4
−CF5
+H5
−CF6
+H6
−CF7
+H7
Potential-determining step (PDS)
2
0
-2
-4
-6
-8
-10

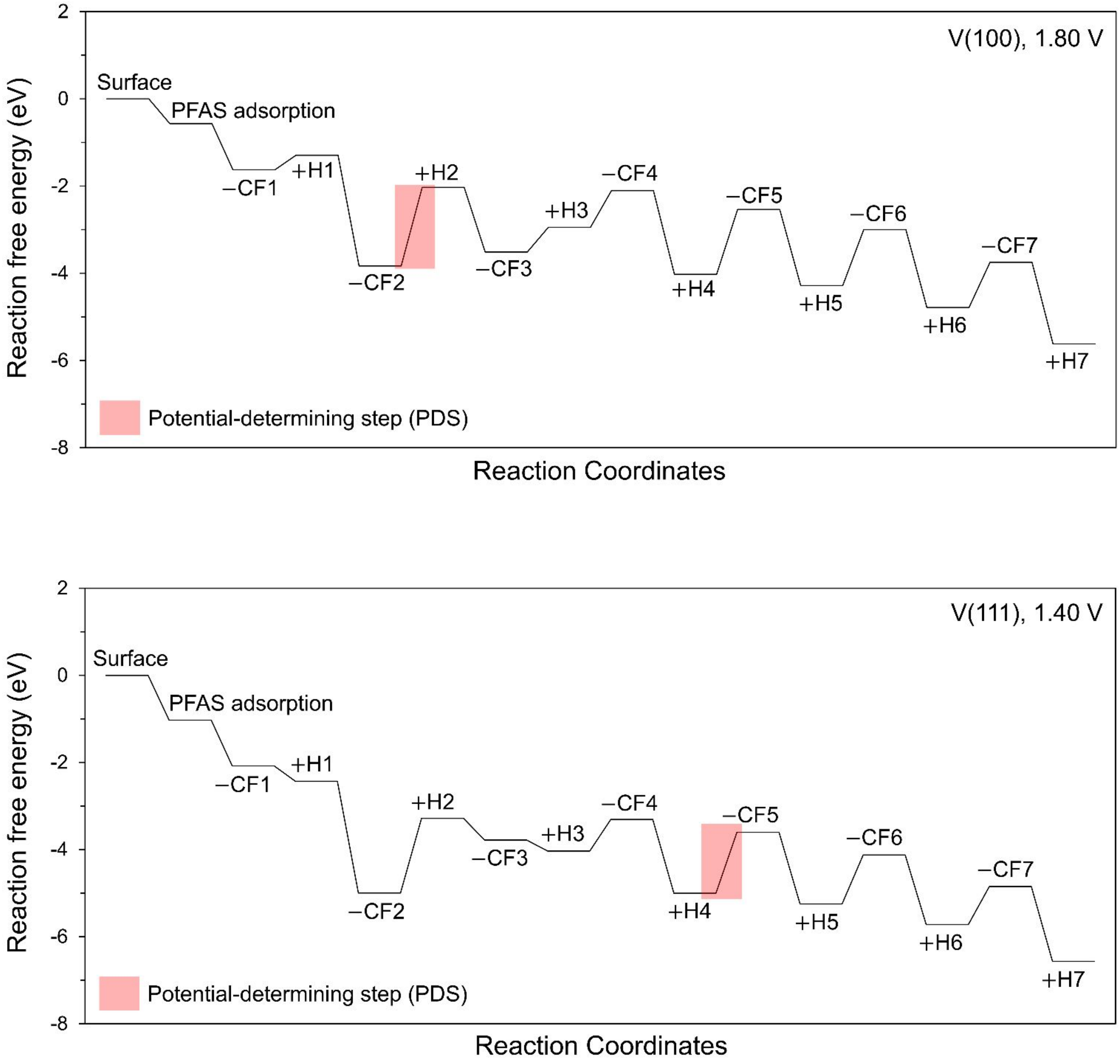
V(100), 1.80 V
Reaction free energy (eV)
Surface
PFAS adsorption
−CF1
+H1
−CF2
+H2
−CF3
+H3
−CF4
+H4
−CF5
+H5
−CF6
+H6
−CF7
+H7
Potential-determining step (PDS)
Reaction Coordinates
V(111), 1.40 V
Reaction free energy (eV)
Surface
PFAS adsorption
−CF1
+H1
−CF2
+H2
−CF3
+H3
−CF4
+H4
−CF5
+H5
−CF6
+H6
−CF7
+H7
Potential-determining step (PDS)
Reaction Coordinates

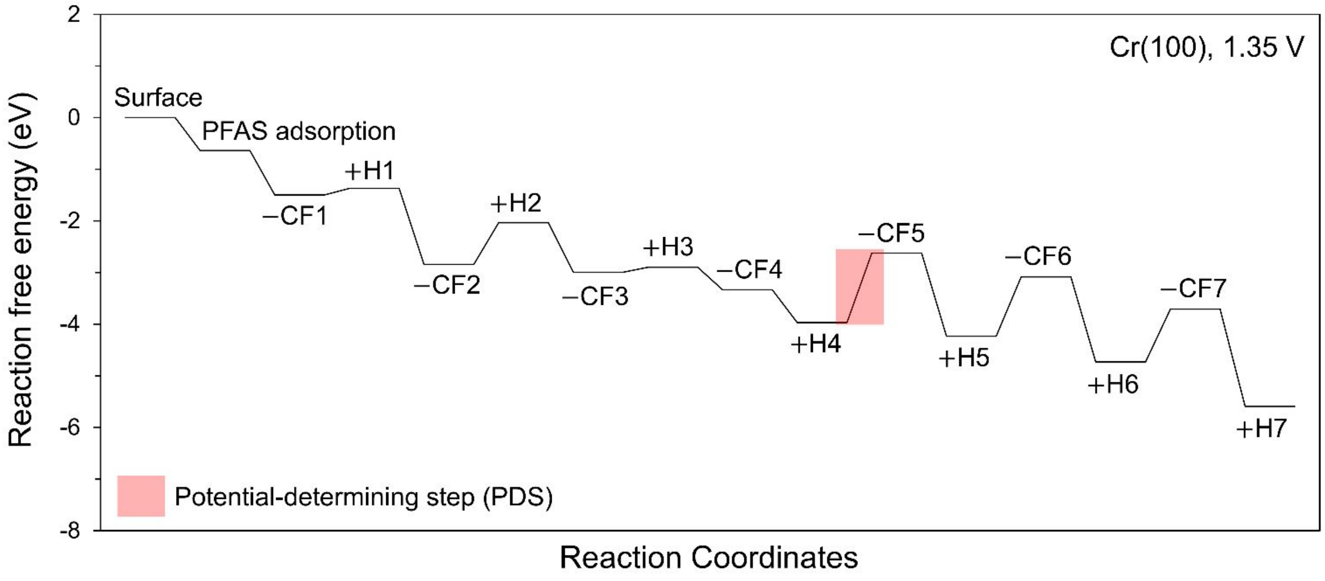
Cr(100), 1.35 V
Reaction free energy (eV)
2
0
-2
-4
-6
-8
Surface
PFAS adsorption
−CF1
+H1
−CF2
+H2
−CF3
+H3
−CF4
+H4
−CF5
+H5
−CF6
+H6
−CF7
+H7
Potential-determining step (PDS)
Reaction Coordinates

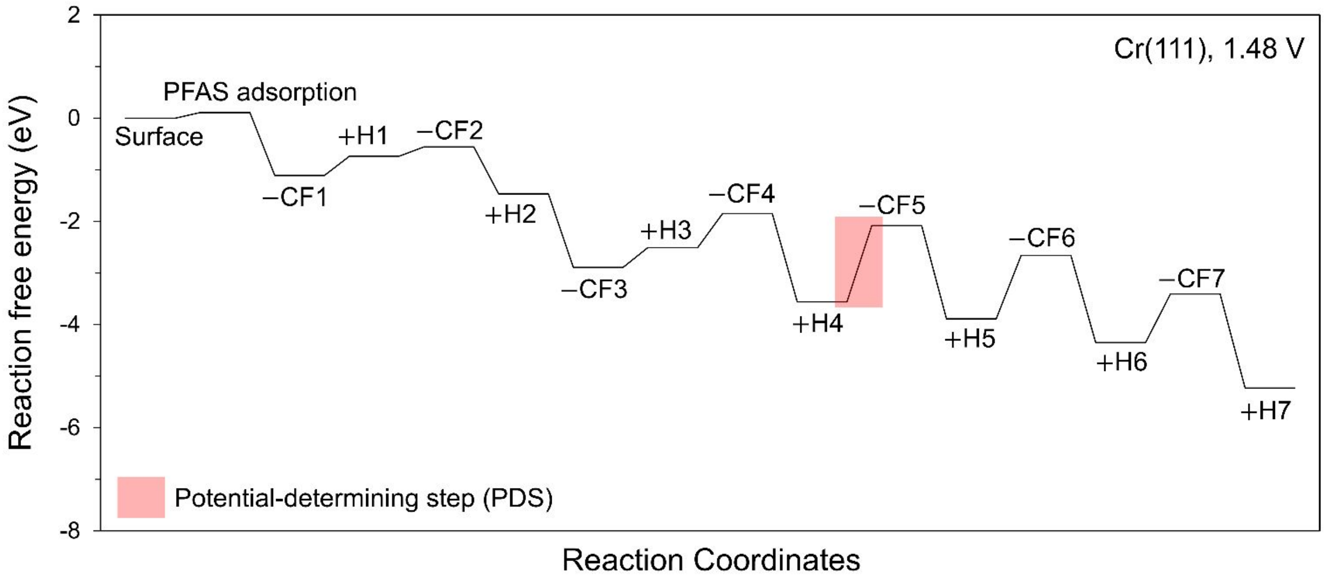
Cr(111), 1.48 V
Reaction free energy (eV)
2
0
-2
-4
-6
-8
PFAS adsorption
Surface
−CF1
+H1
−CF2
+H2
−CF3
+H3
−CF4
+H4
−CF5
+H5
−CF6
+H6
−CF7
+H7
Potential-determining step (PDS)
Reaction Coordinates

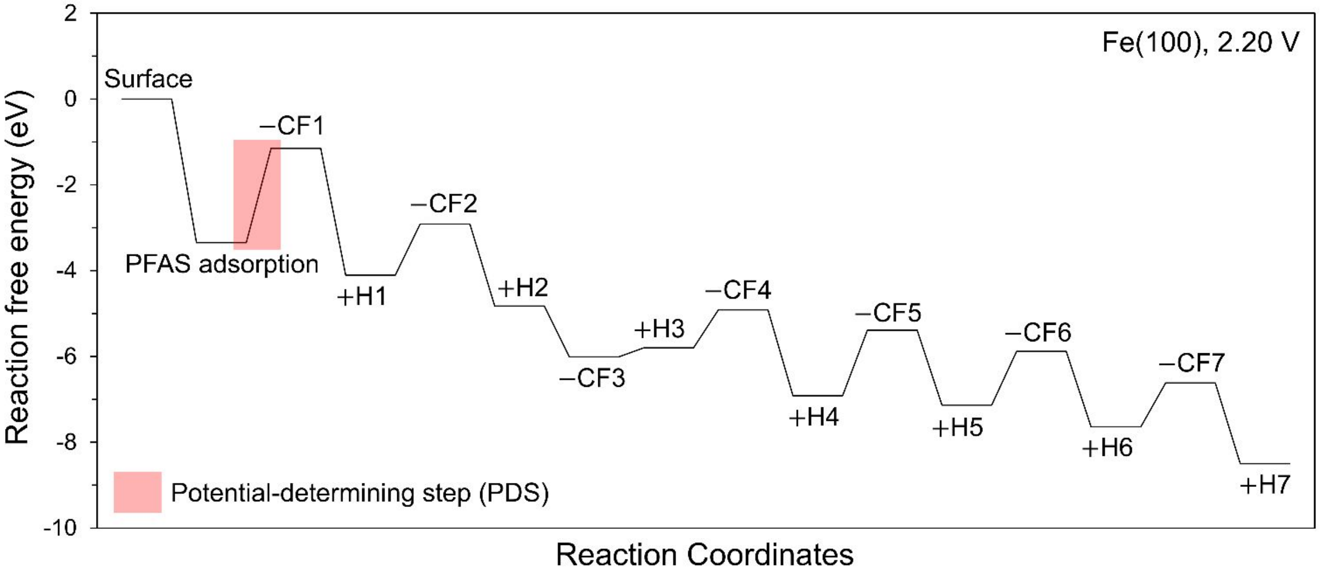
Fe(100), 2.20 V
Surface
PFAS adsorption
−CF1
+H1
−CF2
+H2
−CF3
+H3
−CF4
+H4
−CF5
+H5
−CF6
+H6
−CF7
+H7
Potential-determining step (PDS)
Reaction free energy (eV)
Reaction Coordinates
2
0
-2
-4
-6
-8
-10

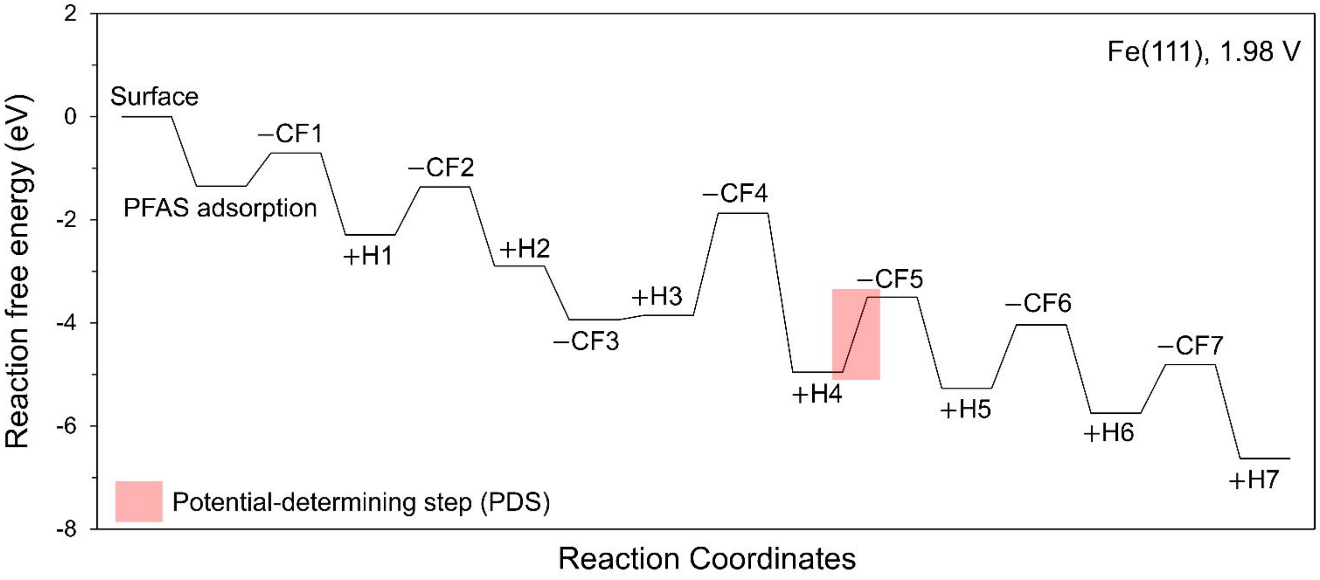
Fe(111), 1.98 V
Surface
PFAS adsorption
−CF1
+H1
−CF2
+H2
−CF3
+H3
−CF4
+H4
−CF5
+H5
−CF6
+H6
−CF7
+H7
Potential-determining step (PDS)
Reaction free energy (eV)
Reaction Coordinates
2
0
-2
-4
-6
-8

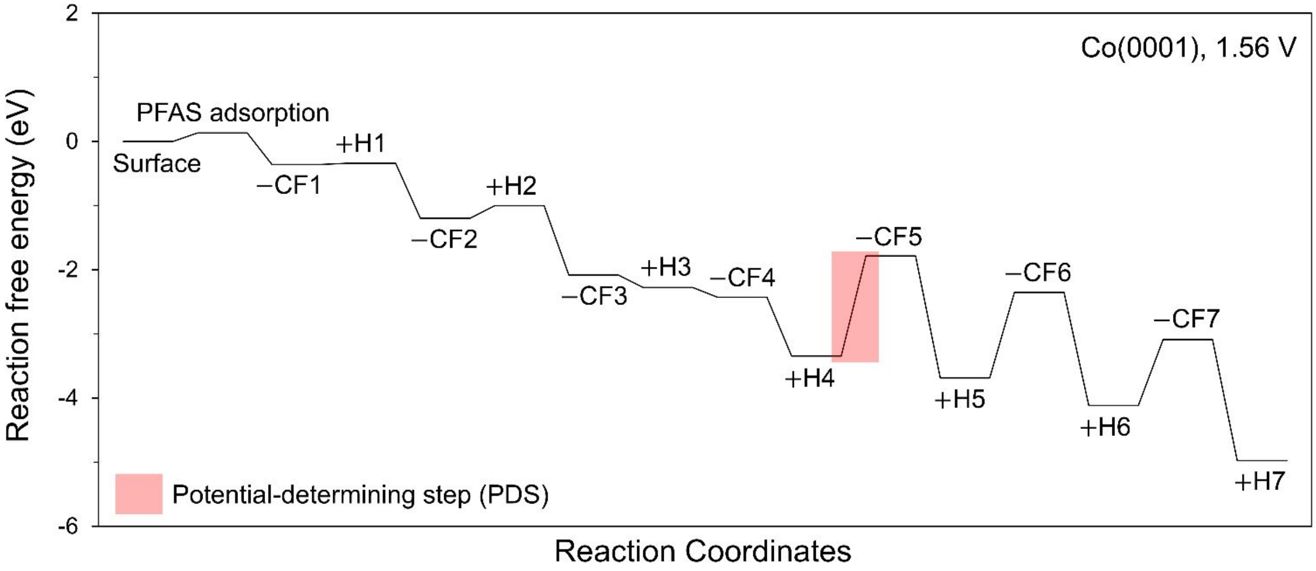
Co(0001), 1.56 V
PFAS adsorption
Surface
−CF1
+H1
−CF2
+H2
−CF3
+H3
−CF4
+H4
−CF5
+H5
−CF6
+H6
−CF7
+H7
Potential-determining step (PDS)
Reaction free energy (eV)
Reaction Coordinates
2
0
-2
-4
-6

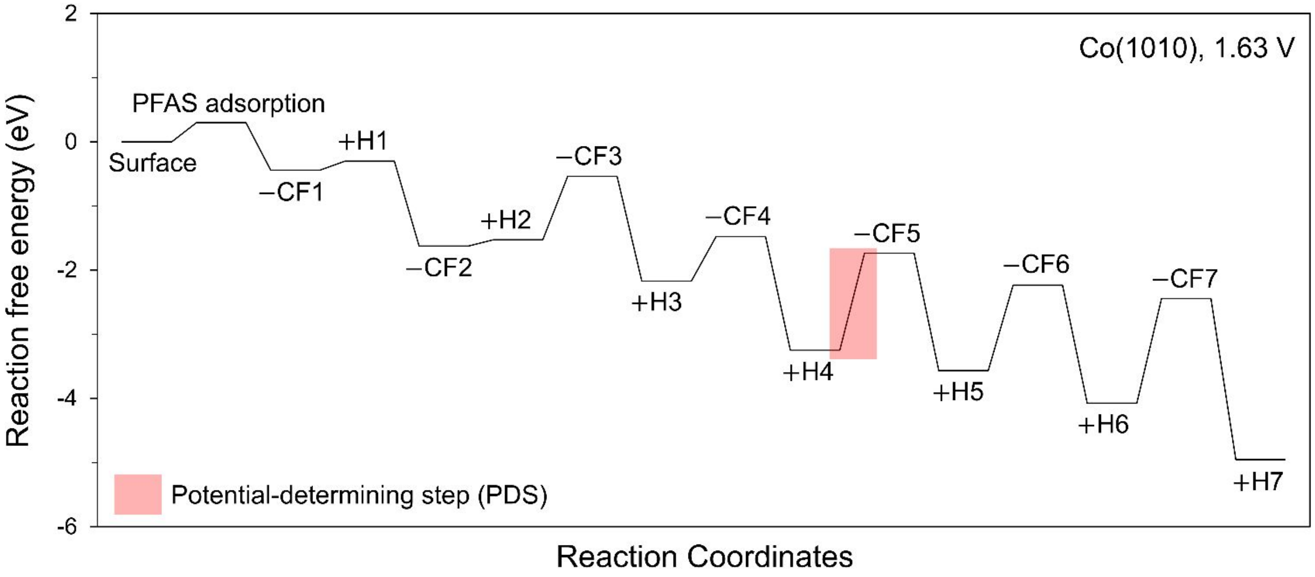
Co(1010), 1.63 V
PFAS adsorption
Surface
−CF1
+H1
−CF2
+H2
−CF3
+H3
−CF4
+H4
−CF5
+H5
−CF6
+H6
−CF7
+H7
Potential-determining step (PDS)
Reaction free energy (eV)
Reaction Coordinates
2
0
-2
-4
-6

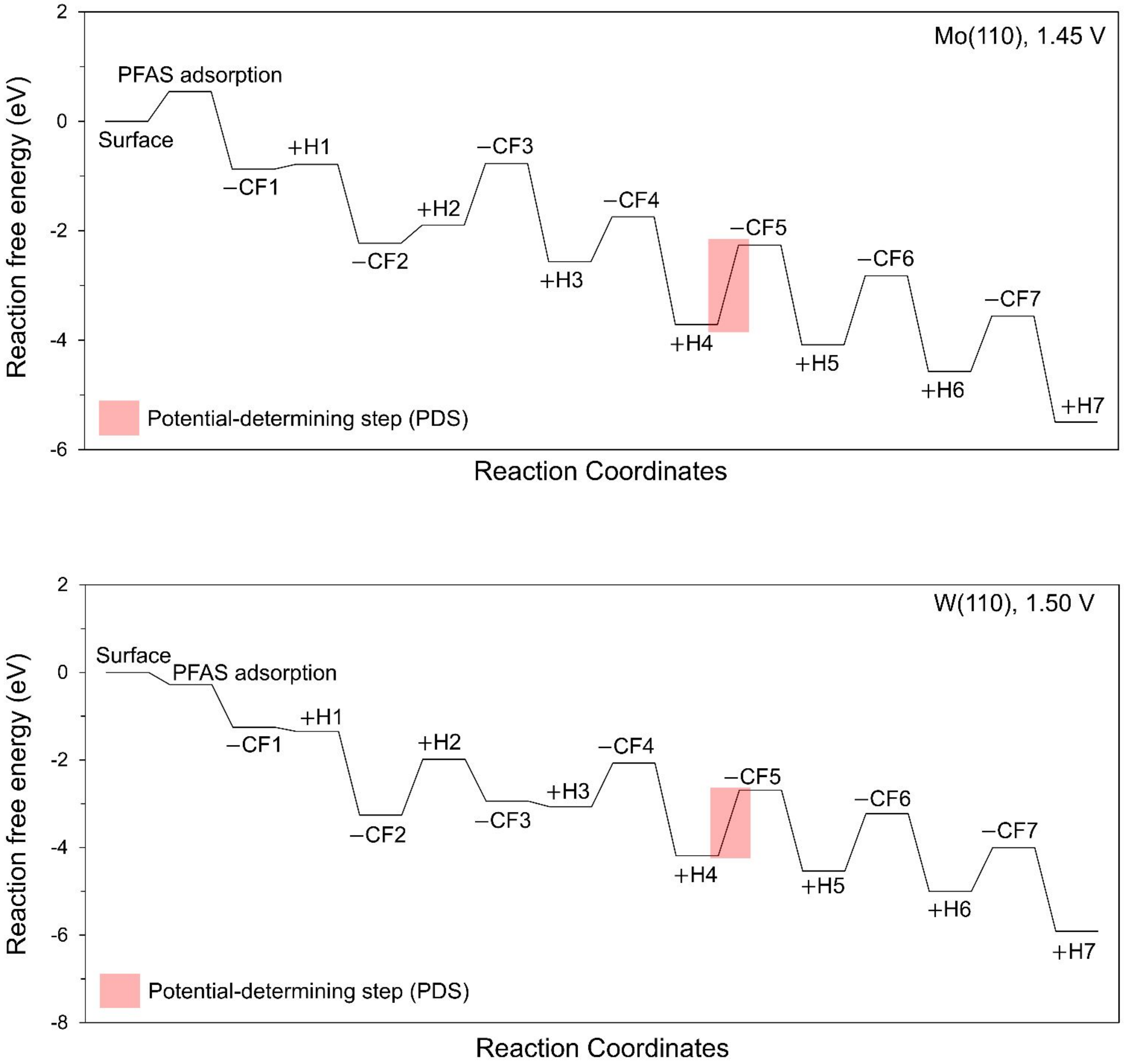

Figure S1. Free energy diagram (FED) for shortlisted 14 TM surfaces along the most favorable pathway: seven C–F cleavages (CF1-CF7) alternate with seven hydrogenations (H1-H7).
